# Exploration of Users’ Perspectives and Needs and Design of a Type 1 Diabetes Management Mobile App: Mixed-Methods Study

**DOI:** 10.2196/11400

**Published:** 2018-09-21

**Authors:** Yiyu Zhang, Xia Li, Shuoming Luo, Chaoyuan Liu, Fang Liu, Zhiguang Zhou

**Affiliations:** 1 Department of Metabolism and Endocrinology The Second Xiangya Hospital Central South University Changsha China; 2 Key Laboratory of Diabetes Immunology, Ministry of Education Changsha China; 3 National Clinical Research Center for Metabolic Diseases Changsha China; 4 Department of Oncology The Second Xiangya Hospital Central South University Changsha China

**Keywords:** diabetes mellitus, type 1, mobile applications, qualitative research, surveys and questionnaires

## Abstract

**Background:**

With the popularity of mobile phones, mobile apps have great potential for the management of diabetes, but the effectiveness of current diabetes apps for type 1 diabetes mellitus (T1DM) is poor. No study has explored the reasons for this deficiency from the users’ perspective.

**Objective:**

The aims of this study were to explore the perspectives and needs of T1DM patients and diabetes experts concerning a diabetes app and to design a new T1DM management mobile app.

**Methods:**

A mixed-methods design combining quantitative surveys and qualitative interviews was used to explore users’ needs and perspectives. Experts were surveyed at 2 diabetes conferences using paper questionnaires. T1DM patients were surveyed using Sojump (Changsha ran Xing InfoTech Ltd) on a network. We conducted semistructured, in-depth interviews with adult T1DM patients or parents of child patients who had ever used diabetes apps. The interviews were audio-recorded, transcribed, and coded for theme identification.

**Results:**

The expert response rate was 63.5% (127/200). The respondents thought that the reasons for app invalidity were that patients did not continue using the app (76.4%, 97/127), little guidance was received from health care professionals (HCPs; 73.2%, 93/127), diabetes education knowledge was unsystematic (52.8%, 67/127), and the app functions were incomplete (44.1%, 56/127). A total of 245 T1DM patient questionnaires were collected, of which 21.2% (52/245) of the respondents had used diabetes apps. The reasons for their reluctance to use an app were limited time (39%, 20/52), complicated operations (25%, 13/52), uselessness (25%, 13/52), and cost (25%, 13/52). Both the experts and patients thought that the most important functions of the app were patient-doctor communication and the availability of a diabetes diary. Two themes that were useful for app design were identified from the interviews: (1) problems with patients’ diabetes self-management and (2) problems with current apps. In addition, needs and suggestions for a diabetes app were obtained. Patient-doctor communication, diabetes diary, diabetes education, and peer support were all considered important by the patients, which informed the development of a prototype multifunctional app.

**Conclusions:**

Patient-doctor communication is the most important function of a diabetes app. Apps should be integrated with HCPs rather than stand-alone. We advocate that doctors follow up with their patients using a diabetes app. Our user-centered method explored comprehensively and deeply why the effectiveness of current diabetes apps for T1DM was poor and what T1DM patients needed for a diabetes app and provided meaningful guidance for app design.

## Introduction

### Background

The incidence of type 1 diabetes mellitus (T1DM) has been increasing worldwide [1,2]. An estimated 13,000 new T1DM cases occur every year in China [3]. Failure of islet beta-cell function occurs in the early stage of T1DM [4]; thus, controlling blood glucose is difficult. Despite the development of therapeutic drugs and treatment techniques, the blood sugar of T1DM patients is still poorly controlled [5]. The 3C study in China showed that the average glycosylated hemoglobin (HbA_1c_) of T1DM patients in Beijing and Shantou was 8.5% [6], far higher than the guideline recommendations [7], and a clear gap existed between China and developed countries. Poor glycemic control can cause various complications [8] and place heavy financial burdens on the country and patients.

For T1DM patients, self-management ability is very important [9]. Increasing communication with doctors and strengthening blood sugar monitoring are beneficial for glycemic control [10,11]. The following challenges are present in outpatient clinics: inconvenience because of time and space limitations; limited ability to gain diabetes self-management knowledge in a short period of time; and compliance with a diabetes diary is often poor, which prevents doctors from providing effective treatment guidance [12]. Due to the imbalance of medical resources in China [13], patients flock to tertiary hospitals in large cities to seek medical resources, but they receive an outpatient consultation lasting just a few minutes. Continuity of care is a challenge in traditional outpatient settings as T1DM patients usually do not return to the same hospital or at regular intervals [6]. Mobile apps can record, transmit, and receive feedback anytime and anywhere. Mobile phones have been integrated into individuals’ personal lives because of their popularity [14]. Thus, an app has great potential for the management of diabetes [15], especially for patients from remote areas.

However, people do not continue using health apps because of data entry burden and loss of interest [16]. Pernille’s study revealed that the use of a diabetes self-management app by young T1DM patients decreased gradually after the first few weeks [17]. The majority of diabetes apps contain only a few functions [18]. The number of functions offered by apps influences HbA_1c_ levels [19]. Diabetes apps achieve different results in terms of glycemic control [15,20]; the effects in T1DM patients are poor [21].

App development must be closely integrated with clinical guidelines, and they must work closely with health care professionals (HCPs) and patients [22]. Most apps are developed by software engineers without medical backgrounds [21]. Thus, the developed apps are not well integrated with guidelines and clinical needs [21,23]. For example, despite the emphasis by diabetes guidelines for the need for ongoing patient education [24], very few studies used mobile apps that have education as a functionality [23]. Personalized education is an under-represented feature in diabetes mobile apps [25], and the role of HCPs is missing in most apps [15].

T1DM is different from type 2 diabetes mellitus (T2DM) in many aspects [26]. For example, T1DM patients are younger. They are insulin-dependent, whereas most T2DM patients do not require insulin treatment. Insulin dose and carbohydrate calculation and self-monitoring of blood sugar are more important for T1DM patients. However, although numerous diabetes apps have been developed, few are specific for T1DM [27]; thus, the developed apps might not be suitable for T1DM patients.

Few diabetes apps have been introduced with the methodology of their development [23]. Gaining a deep understanding of the perspectives of patients is important when developing a mobile app for their use [28,29]. Qualitative research methodology has become more recognized and valued in diabetes behavioral research. By exploring patients’ motivations, perspectives, and expectations, this approach can answer questions that cannot be addressed using a quantitative study. A mixed-methods study can combine qualitative and quantitative results to provide a more comprehensive and deeper understanding of user perspectives [30].

### Objectives

No study has explored the reasons for poor effects of current diabetes apps in T1DM patients from the users’ perspectives. To improve glycemic control in Chinese T1DM patients, we used a mixed-methods study to explore users’ perspectives and needs and cooperated with a software team to develop a mobile app for T1DM management.

## Methods

### Part 1: Questionnaire Survey

#### Questionnaire Design

An expert panel consisting of 3 diabetologists (YZ, SL, and XL) and a diabetes education nurse (FL) from our hospital designed the questionnaires according to the functions of current diabetes apps [18,21,25,31-33], the problems they encountered during clinical practice, and diabetes guidelines [26]. The questions were presented in a choice format. If responders did not agree with the listed options, they could select the option “other” and write their answers in the remarks column. The expert questions covered their use of and perspectives about diabetes apps. The patient questions covered their use of, perspectives about, and needs for diabetes apps; demographic information; and basic disease information. Before the questionnaires were administered, we performed pilot tests with 10 diabetologists in our hospital and 20 diabetes patients from our outpatient department.

#### Samples and Survey Methods

The expert questionnaires (Multimedia Appendix 1) were administered using a paper format at 2 national diabetes conferences held in October 2017 and December 2017, with a total of 200 diabetologists attending. From 23rd January to 1st March 2018, the T1DM patient questionnaires (Multimedia Appendix 2) were administered using the Web-based questionnaire tool Sojump on the WeChat network [34]. The questionnaire links were spread among the first author’s WeChat friends circle and WeChat groups of diabetes patients. The questions were answered by adult patients or the parents of child patients. No compensation was given for participation in the study.

#### Data Analysis

Descriptive statistics were used to characterize the samples. Frequencies and percentages were used to describe categorical variables. Incomplete responses were included in the analysis.

### Part 2: Qualitative Study

#### Data Collection

After administering the questionnaire surveys, semistructured one-on-one in-depth interviews were conducted by a diabetologist (YZ). T1DM patients who had previously used diabetes apps were contacted. First, we introduced the objective of the study to establish trust. Adult patients or the parents of child patients were invited for a one-on-one interview. The interview environments were quiet, and interruptions were minimized. An interview guideline (Multimedia Appendix 3) was created by the expert panel and covered questions about the patients’ daily diabetes management behavior, problems with apps they had used in the past, and their needs and suggestions for an app. The questions were open-ended. Each interview lasted approximately 30 to 60 min. Data collection ended when data saturation was achieved [35]. All interviews were audio-recorded, and all participants gave written informed consent.

#### Data Analysis

The data analysis was ongoing during the data collection process to ensure data saturation. Records were transcribed verbatim by the interviewer (YZ) and were verified by the interviewees. Data analysis was managed using NVivo 11.0 (QSR International Pty Ltd). Using inductive thematic analysis [36], the transcripts were independently read and coded by 2 investigators (YZ and XL). Disagreements and emerging themes were discussed with the expert panel.

### Part 3: App Prototype Design and Development

On the basis of the results of the questionnaires and interviews, the expert panel combined their clinical experiences and clinical guidelines [9,24,26] to determine the modules and contents of the app and held discussions with the software team at least once a week in the form of workshops. The software team developed the app iteratively using an agile software development methodology. During each workshop, the software team introduced their app design and the prototype developed in the last iteration. The expert panel operated the prototype and proposed some modifications and new requirements for the app according to their expertise. One patient was invited to share their user experience in each workshop. The workshop members discussed the layout, design, and contents of the app. Brainstorming was adopted in this process. The software team developed the app accordingly in the next iteration.

### Ethical Approval

The study was approved by the ethics committee of the Second Xiangya Hospital, Central South University.

## Results

### Part 1: Questionnaire Survey

#### Expert Survey

##### Factors Influencing Experts’ Use and Recommendation of Diabetes Management Apps

**Figure 1:**
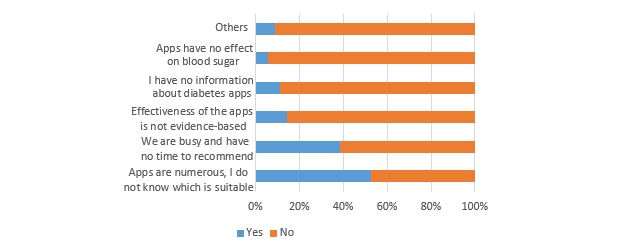
Factors influencing experts’ recommendation of diabetes apps (n=127).

**Figure 2:**
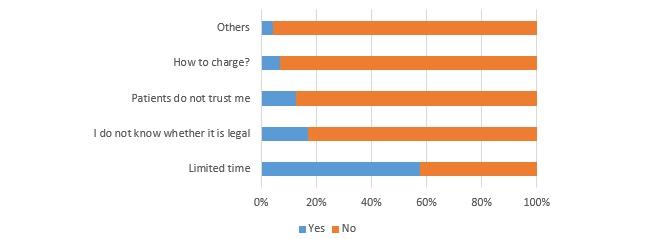
The biggest obstacles to experts’ use of apps to manage diabetes patients (n=118).

The response rate for the expert survey was 63.5% (127/200). Overall, 52.8% (67/127) of the experts had recommended diabetes apps to their patients. Figure 1 shows the factors influencing their recommendations for a diabetes app. A total of 34.6% (44/127) of the experts had used diabetes apps to manage diabetes patients. These experts thought that the biggest obstacle to their use of apps to manage diabetes patients was limited time (57.6%, 68/118; see Figure 2). A total of 57.5% (73/127) of the experts did not know whether using an app to manage patients was legal, 26.7% (34/127) thought that using an app for this purpose was legal, and 15.0% (19/127) thought that it was illegal.

##### Experts’ Perceptions of Diabetes Management Apps

The experts’ proposed reasons for app invalidity were that patients did not continue using them (76.4%, 97/127), patients received little guidance from HCPs (73.2%, 93/127), diabetes knowledge on the app was unsystematic (52.8%, 67/127), and the apps’ functions were incomplete (44.1%, 56/127). The experts thought that the most important functions of an app were patient-doctor communication (42.4%, 53/125), the diabetes diary (39.2%, 49/125), diabetes education (10.4%, 13/125), and abnormal blood sugar reminders (6.4%, 8/125). Most experts did not recommend or were opposed to insulin calculators (62.0%, 75/121) because 78.2% (97/124) thought that these tools were dangerous or very dangerous. Overall, 82.5% (104/126) of the experts thought that the prospect for diabetes apps was good or very good.

#### Patient Survey

##### Factors Influencing Patients’ Use of Diabetes App

A total of 245 T1DM patient questionnaires were collected. Table 1 shows the characteristics of the respondents. Overall, 61.2% (150/245) of the responders did not know about the existence of diabetes apps, and only 21.2% (52/245) had ever used diabetes apps. Only 8% (4/52) of the apps were recommended by HCPs. Most of the apps were recommended by patients (38%, 20/52) or selected randomly (37%, 19/52) because the respondents did not know which app was the best.

**Table 1:** Characteristics of patients responding to the surveys.

Characteristics		Total (N=245)	Adolescent (n=115)	Adults (n=130)
**Gender, n (%)**				
	Male	98 (40.0)	49 (42.6)	49 (37.7)
	Female	147 (60.0)	66 (57.4)	81 (62.3)
Age in years, median (IQR^a^)		18 (11-30)	11 (8-14)	29 (23-35.3)
Disease duration in years, median (IQR)		3 (1-9)	2 (1-4)	5 (1.75-15)
**Treatment type, n (%)**				
	Insulin pump	65 (26.5)	27 (23.5)	38 (29.2)
	Insulin injection	280 (73.5)	88 (76.5)	92 (70.8)

^a^IQR: interquartile range.

**Figure 3:**
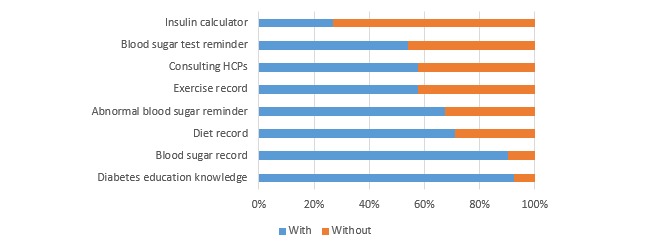
Proportions of different functions of patients’ diabetes apps (n=52). HCP: health care professional.

The reasons for their reluctance to use an app were limited time (39%, 20/52), complicated operations (25%, 13/52), uselessness (25%, 13/52), and cost (25%, 13/52). The most common functions of their apps were diabetes knowledge (92%, 48/52) and blood sugar record (90%, 47/52; see Figure 3). A total of 70% (33/47) of the patients thought manual input of blood sugar was troublesome or a little troublesome. A total of 58% (30/52) of the apps could consult HCPs, but only 30% (9/30) of the patients had ever used this function.

##### Patients’ Needs for a Diabetes App

**Figure 4:**
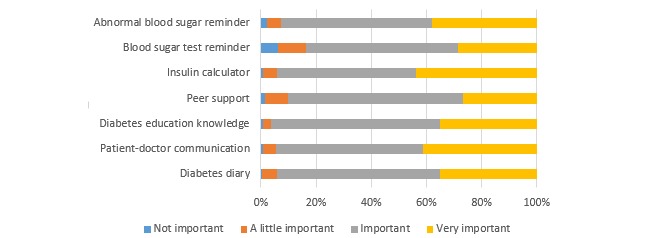
Usefulness of app functions reported as important by patients (n=245).

The patients thought the most important functions of the apps were consulting HCPs (33.9%, 83/245), the diabetes diary (24.4%, 55/245), diabetes knowledge (12.7%, 31/245), the insulin calculator (11.8%, 29/245), abnormal blood sugar reminders (10.6%, 26/245), peer support (2.9%, 7/245), and blood sugar test reminders (1.2%, 3/245). Almost all patients thought the above functions were important or very important (see Figure 4). A total of 65.3% (160/245) of the patients thought that they were in need or in great need (32.7%, 80/245) of a good app to manage their diabetes.

### Part 2: Qualitative Study

#### Participants

The final sample consisted of 18 participants (12 adult patients and 6 parents of young patients; see Table 2).

#### Themes

Two themes including 10 subthemes that were helpful for our app design were identified.

##### Theme 1: Problems in Patients’ Diabetes Self-Management Conduct

Diabetes self-management education (DSME), diet, exercise, and self-monitoring of blood sugar are 4 important parts of diabetes self-management. Understanding the problems with self-management helped refine the design of our app.

###### Diabetes Self-Management Education

Most patients did not receive DSME programs in the hospital. DSME in the hospital had many shortcomings, including inconvenience, reluctance of young people to go to the hospital, lack of individualization, and low efficiency. Compared with receiving DSME in the hospital, receiving information on mobile apps was preferable. The patients could select subjects that they were interested in, learn repeatedly, and learn when they had time. Additionally, the time and economic costs were lower.

Two patients stated the following:

From Monday to Friday, there is no time. Secondly, I think sometimes we will select contents to learn after we have mastered some knowledge. Because we have mastered some basic knowledge, if lectures are about such contents, we will not go to learn.

P5, 30-year-old female

Both are fine. But if I go to the hospital, I feel I have no time. Because if I learn on a mobile app, videos can be saved; I can learn when I have time. I think the app is better.

P10, 24-year-old female

**Table 2:** Characteristics of the interviewees.

Characteristics		Adult patients (n=12)	Parents of young patients (n=6)
**Gender, n (%)**			
	Male	1 (8)	3 (50)
	Female	11 (92)	3 (50)
Patients’ age in years, mean (range)		26.8 (20-33)	10.5 (6-16)
Patients’ disease duration in years, mean (range)		4.0 (1-12)	3.3 (0-9)
**Treatment type, n (%)**			
	Insulin pump	3 (25)	3 (50)
	Insulin injection	9 (75)	3 (50)
**Education, n (%)**			
	Postgraduate	1 (8)	—
	University	8 (67)	4 (67)
	High school	3 (25)	1 (17)
	Junior middle school	—	1 (17)
**Residence, n (%)**			
	Urban	9 (75)	5 (83)
	Rural	3 (25)	1 (17)

###### Self-Monitoring of Blood Sugar

Space, time, economy, and pain were all factors that influenced blood sugar tests. Some patients forgot to test because they did not form a habit of testing their blood sugar, or they were doing other things. Some patients did not know when they needed to test, and some were not aware of the importance of testing. One patient stated the following:

It is not as important as insulin injection. If you don’t inject insulin, your blood sugar will surely be high. But if you pay attention to your food, you have a sense of your blood sugar level, so you don’t attach much importance to it...

P11, 31-year-old female

###### Diet

Some patients had incorrect diet conceptions. Calculating carbohydrates and calories is important for blood sugar control, but most patients do not perform these calculations for their daily diets. They thought that the calculation process was complicated and troublesome. One patient stated the following:

I don’t know. At the beginning, they told me to calculate. It is complicated. In a WeChat group, some people told me how to calculate, and when I came to the nutrition department, they told me how to calculate. But after that I will say, I try to eat vegetables as much as possible.

P15, 27-year-old female

###### Exercise

Most patients knew the importance of exercise for glycemic control, but many of them lacked the time and will. Some patients selected the wrong time to exercise. Some were afraid to exercise because they were worried about hypoglycemia, as illustrated in the following quote:

Blood sugars fluctuate greatly. I dare not exercise. I’d rather have higher blood sugar. I’d rather give a bolus. I’m not willing to exercise.

P12, 26-year-old male

##### Theme 2: Problems With the Functions of Current Apps and Patients’ Needs and Suggestions for a New App

###### Diabetes Diary

Although they thought a mobile diabetes diary was more convenient than a paper diary, most of them thought manual input was burdensome (see Table 3 for the problems with current diabetes app). The patients wanted glucose data to be transmitted to the apps automatically. Diet and exercise records were even more troublesome. Some of the patients thought that these types of records were useless and that their display was not as intuitive as that of a paper diary. Most of the patients only recorded blood sugar.

One patient stated the following:

If input manually, when you are outside, testing blood sugar is inconvenient, but you have to record...you will think it doesn’t matter. They are just in the glucose meter. It’s very burdensome. But if it can be transmitted to app automatically, it is convenient.

P7, 33-year-old female

Most patients reported that the greatest problem with diabetes diaries was the lack of feedback from HCPs. As the diary was useless for glycemic control, they did not continue to use the apps. They hoped to obtain feedback after recording and to have a doctor analyze their data.

**Table 3:** Problems with current diabetes apps and needs for a new app.

Modules of current app	Problems and needs
Patient-doctor communication	Distrust Responses are not timely Inconvenient Cost
Diabetes diary	Burdensome Lack of feedback Display is not as intuitive as a paper diary Food database is needed
Diabetes education knowledge	Unsystematic Unprofessional Avoid irrelevant knowledge interference Find materials of interest easily Update in a timely manner Interaction is needed Tend to learn pop-up knowledge Different learning habits
Peer-support	Inconvenient Avoid excessive information interference Peer leader is needed Privacy protection
Psychological module	Most apps lack this module
Electronic health records	Access to hospital electronic medical records

One parent stated the following:

It is meaningless if you record there. But if these data, I think, let me think, if after these data are submitted, an online doctor analyzes them for you, I think people will like it.

P6, mother of a 10-year-old patient

###### Patient-Doctor Communication

Some diabetes apps had a function for consulting HCPs. However, most users did not consult HCPs using the app because they did not trust unfamiliar doctors. App communication in the form of typing words was inconvenient, and the communication efficiency was low. Consultations needed to be charged, feedback was not timely, and the consultation effect was low. These factors hampered consultations with doctors by the patients using the apps.

Two patients stated the following:

I tried once to make an appointment with a doctor in the weltang app. But for his few minutes he needed to charge, so I exited. An unfamiliar doctor, you consult him, but you need to pay. Maybe you have a sense of...

P11, 31-year-old female

I consulted once. Because the doctor was busy, the response was not timely. Describing our condition by typing words, maybe it is not so good to meet the needs of patients. After all, they are not our familiar doctors, they don’t know our condition. I hope to communicate directly with the doctor.

P4, 30-year-old female

Most patients want to consult doctors on the app. However, doctors approached via the internet are not familiar with the patients’ conditions. The patients wanted their outpatient doctors to continue to follow them up. Doctors from primary hospitals lack experience with managing T1DM. Moreover, the patients do not trust doctors from primary hospitals and only trust doctors from large tertiary hospitals. One patient stated the following:

Yes, unless he is your outpatient doctor. I think it can be set on that app, for example, you consult your outpatient doctor and have good effects.

P7, 33-year-old female

One parent stated the following:

There are only two type 1 diabetes patients in our county. When I went to the county hospital to ask the doctors, they never heard of this disease...

P8, father of a 12-year-old patient

###### Diabetes Education

Most patients hoped to gain diabetes knowledge on the app. They were most concerned about the latest progress in diabetes, knowledge about complications, nutrition, exercise, and insulin dose calculation. Some patients thought diabetes knowledge on apps was unsystematic and unprofessional. Patients did not know whether the diabetes knowledge was accurate. Patients hoped for the inclusion of authoritative and practical knowledge. One patient stated the following:

It’s too miscellaneous. You can’t tell which is right. Because now most of us get information through the internet, I think accuracy is important for information about disease.

P7, 33-year-old female

The patients liked different modes of educational materials. Some liked to watch videos, whereas others liked to read articles. They hoped diabetes knowledge could be classified according to categories and that knowledge about T1DM could be separated from that about T2DM, which would enable the patients to learn pertinent information and avoid excessive information interference, as illustrated in the following quote:

Because I’m type 1, so it is more targeted...we are all type 1. It is not mixed with type 2. Because other apps were mixed with type 2 diabetes, gestational diabetes, and so on, it’s really very chaotic. There is lots of information. You need to screen which is useful, which is useless.

P7, 33-year-old female

###### Peer Support

Almost all the patients wanted to communicate with similar diabetes patients. Some patients said they had no way to find such patients after the onset of diabetes. They thought peer support could help them exchange glycemic control strategies and emotional experiences. Some of them even thought that patient experiences were more important than consulting doctors because patient experiences were person-specific and practical, as illustrated in the following quote:

There are a lot of these patients in our group. Their disease durations are many years. Their own experiences may be better than that of doctors because they are more practical. What the doctor said is theoretical. Some diabetic friends, based on their own experiences, may be more practical.

P6, mother of a 10-year-old patient

Many patients believed that having a peer leader was very important. Patients with a long disease duration and rich experience in glycemic control can act as peer leaders. Peer leaders can play a leading, interactive, and cohesive role and drive the atmosphere of a peer support module, as illustrated in the following quote:

For example, the key is, like a family, there is no backbone. There is no person with comprehensive knowledge. His knowledge is comprehensive; whatever questions you put forward, he can help you to solve it. Like that teacher, his prestige is high. He is willing to listen to others, and then he is willing to help others.

P8, father of a 12-year-old patient

The patients hoped to have different types of peer support modes. However, all peer communications in the diabetes apps took place in the form of forums. Most patients thought that communicating in that way was inconvenient and that responses were not timely. Very few patients chatted in the diabetes apps, as illustrated in the following quote:

[WeChat] Group chat is timely. Questions you ask can be answered immediately. But on the forum, you will wait a few days. I think feedback in group chat is more timely. It is better. I don’t use forums now...

P5, 30-year-old female

###### Psychological Module

Mental health specialists are recommended as a part of diabetes management by diabetes guidelines. Almost all patients said diabetes brought negative emotions to them to varying degrees. Some patients indicated that the apps had no psychological module, and they hoped we could pay attention to their mental health, as illustrated in the following quote:

Another is psychological, a psychological module for patients. I have lots of apps on my mobile phone. Almost all are about knowledge, how to control blood sugar. Attention to children's mental health, a psychological module doesn’t exist.

P8, father of a 12-year-old patient

###### Electronic Medical Records

The patients hoped to access their hospital electronic medical records (EMRs) through the app (eg, to view their test results and their diagnostic and treatment records and to register for outpatient visits). This possibility would be convenient, allow them to build health records in the app, and motivate them to continue using the app. One patient stated the following:

Connect to hospital health records systems directly. You can register for outpatient visits, and whenever you have problems, you can consult your outpatient doctor. Maybe these can be included.

P5, 30-year-old female

### Part 3: App Prototype

**Figure 5:**
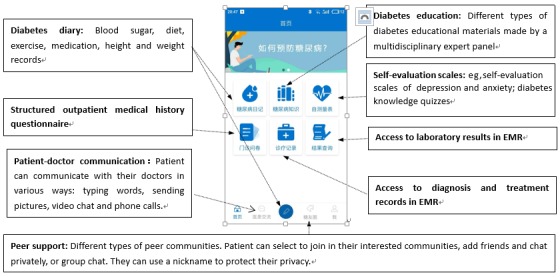
Homepage screenshot of the patient app. EMR: electronic medical record.

The final solution consists of a patient-end app and a doctor-end app. Both of them are based on the iOS and Android platforms. Modules of the patient app are shown in Figure 5.

All the following functions are included: patient-doctor communication, diabetes diary (blood sugar, diet, exercise, and medication records), diabetes education, peer support, blood sugar test reminder, and abnormal blood sugar reminder. According to the expert panel’s clinical experiences, it is important to know patients’ former diagnosis and laboratory results if they are to give treatment recommendations for their patients, and the qualitative results suggested that access to EMRs would motivate patients to continue using the app. This function is also included in our app.

The qualitative results suggested that inconvenience and lack of a timely response were problems with the patient-doctor communication function of current apps, and according to our expert panel’s clinical experience, it is difficult for patients to describe their condition clearly just by typing words. Thus, patient-doctor communication in our app employs various types of communication modes to ensure this convenience: typing words, sending pictures, video chat, and phone calls. Doctors can view their patients’ diabetes diaries, diagnosis, treatment records, and laboratory results in their EMRs and the lengths of patients’ study times with the diabetes education materials through their own app and can give tailored feedback or send tailored education materials to them. Notifications are automatically sent to patients or their doctors if there are new messages for them.

Data entry burden, lack of feedback, and a display that is not as intuitive as a paper diary discourage patients from maintaining diabetes diaries. According to our solution, patients can link the app with their glucose meter, and blood sugar results can automatically be transferred from glucose meters to the app by Bluetooth or General Packet Radio Service. Our app obtains daily step count data from step counter software in mobile phones and records the daily step counts automatically. Patients can take photos of foods to record their diet using the built-in camera. Due to the limitation of the mobile phone page, displays of blood sugar, diet, exercise, and medication records in most apps are scattered across different pages, and it is inconvenient to combine them together. By brainstorming, we constructed a design that enables specific diet, exercise, and medication information to be viewed on the blood sugar display page, thus allowing a comprehensive analysis of the causes of blood sugar changes. Blood sugar history graphs and statistics make blood sugar clear at a glance. For feedback on the diabetes diary, we included the following solutions: (1) blood sugar targets can be established collaboratively by patients and their doctors; (2) patients are alerted to off-target blood sugar with warning colors and messages; (3) if blood sugar levels are dangerous (lower than 3.9 mmol/L or higher than 20 mmol/L), a reminder message will be sent automatically to the patient’s doctor’s app; and (4) a patient’s doctor can view the patient’s diabetes diary and give tailored feedback.

More information about our app functions and design based on quantitative and qualitative results is shown in Multimedia Appendix 4.

## Discussion

### Principal Findings

Our study established the reasons that the effects of current diabetes apps for T1DM are poor and investigated patient requirements from the users’ perspective.

The questionnaire surveys suggested that patient-doctor communication and the availability of a diabetes diary were the most important functions of a diabetes app. Detailed records of blood sugar, diet, exercise, and medication can help doctors analyze the factors influencing blood sugar. The blood sugar record was the most used function of the apps [37], but the users did not continue to use this feature. The in-depth interviews revealed that the greatest problem with the diabetes diaries was the absence of feedback from HCPs. Automatic feedback could not meet patients’ needs. Patients thought that the diary was useless; thus, they gradually stopped using it. Most experts thought 1 important reason for app invalidity was that patients received little guidance from HCPs. Our study suggests that the role of the doctor is central for a diabetes app. A recent meta-analysis revealed that the effects of diabetes apps were explained by the frequency of HCP feedback. HCP functionality is important for achieving clinical effectiveness [38], but few apps offer an integrated function for communication and feedback from HCPs [39]. The questionnaire surveys showed that some diabetes apps had an HCP consultation function, but only a small number of patients had used this function. Through in-depth interviews, we identified the reasons for patients not using this function. One important reason was that patients did not trust unfamiliar doctors on the app, and doctors from primary hospitals in China lacked experience in managing T1DM patients.

We advocate that doctors follow up with their outpatients or inpatients using this app. Internet hospitals are developing rapidly in China. A cross-sectional survey determined that 43 internet hospitals were established in 2017, and patients accessed outpatient service delivery via app in 43% of these hospitals [40]. However, doctors from primary hospitals in China need training to enhance their expertise [41]. Many doctors did not know whether using an app to guide patients’ medication was legal, and doctors in China are overloaded [42]. These issues discourage doctors from using an app to manage patients. Health insurance coverage and charge systems are also needed to encourage HCPs to use an app to manage their patients in the long term. Fortunately, standardization of residents’ training and the hierarchical medical system is underway in China, which will reduce the burden of doctors from tertiary hospitals and will enhance the expertise of doctors from primary hospitals. The Chinese Government is energetically advocating internet medical treatment [43], which will enable doctors to follow up with their patients using an app.

DSME is an important part of diabetes management according to diabetes guidelines. Several studies have shown the benefits of DSME [24]. However, few patients in China receive DSME programs in hospitals [6]. Our in-depth interview found some problems in patients’ self-management conduct and suggested that a mobile app was preferable to education in a hospital for DSME. Digital health interventions can help overcome some of the barriers to self-management posed by the limitations of existing health care systems [44]. The questionnaire surveys suggested that both experts and patients thought DSME was very important for a diabetes app. The experts believed 1 important reason for app invalidity was that diabetes education knowledge on apps was unsystematic. Many diabetes apps do not have sound educational quality [27]. Different modes of systematic diabetes education knowledge created by a multidisciplinary expert panel are needed for the app.

The effectiveness of peer support for diabetes outcomes is ambiguous because of the availability of different support modes [45]. Our study showed that most patients thought peer support was an important function of a diabetes app, and most patients hoped to communicate with similar patients. Peer support can help patients exchange glycemic control strategies and emotional experiences. They considered the role of peer leaders very important. Peer leaders can play leading, interactive, and cohesive roles and can improve the atmosphere of a peer support module. Internet-based mentoring programs can increase the frequency of blood sugar monitoring [46], and studies have demonstrated that peer leaders can provide effective diabetes self-management support [47,48]. However, exchanges in current diabetes apps all take the form of forums, which is inconvenient. Thus, few patients exchanged information with others in diabetes apps.

The expert survey suggested that 1 important reason for app invalidity was that diabetes apps lacked comprehensive functions. A meta-analysis revealed that the number of functions offered by apps influences HbA_1c_ levels [19]. Therefore, modules such as patient-doctor communication, diabetes diary, diabetes education, and peer support are all included in our app. However, diabetes apps offering a wider range of functions performed worse in terms of usability [49], and our study suggested that lack of time and complicated operations were factors influencing patients’ use of an app. Most patients considered the manual input of diabetes diary data burdensome. To increase app usability and patients’ adherence to complete a diabetes diary, blood sugar readings and daily steps can be recorded automatically in our app. Of course, feedback from HCPs will encourage patients to adhere to diabetes diaries. Our app design principle was that the operations should be simple and clear, and the use of clear navigation in our app will enable its usability.

The patient survey suggested that patients greatly needed an insulin calculator, but they did not know whether the calculator was accurate. The expert surveys suggested that most experts did not recommend or were opposed to an insulin calculator, and most of them thought insulin calculators were dangerous or very dangerous. Similar results were found in a New Zealand survey [37]. As these algorithms were found to have limited efficacy and were incorrect [50], we did not include an insulin calculator in our app. Artificial intelligence may have potential use in this area [51].

Our study revealed that the awareness and utilization rates of diabetes apps in China were low. Only a small subset of the patients’ apps was recommended by HCPs. One important reason was that the effects of the apps were not evidence-based; thus, they did not know which app was better. Only 1 Chinese diabetes app was tested in a short-term randomized controlled trial (RCT) [52]. Thus, high-quality RCTs are needed [39]. We are planning a multicenter RCT to test the long-term efficacy of our app. We hope we can provide evidence for patients to choose a valid diabetes app.

### Limitations

We did not interview child or adolescent patients as their needs and diabetes management models are slightly different from those of adults. However, in child patients, disease management is always performed by their parents; thus, our app is also suitable for this population. However, children occasionally manage their disease independently. In particular, adolescents of transitioning age gradually withdraw support from their parents and take over management tasks. Our app can help these patients through this transitioning period. We can set a family member account to supervise them, and we included some peer communities of their age in our app to help them solve problems specific to this period. However, further improvement is needed to satisfy the specific needs of this population. We also did not interview diabetes experts. As our expert panel consisted of diabetes experts with rich experience in T1DM management, we did not think that interviewing diabetes experts was necessary. The security of young HCPs regarding making incorrect medical suggestions should be taken into consideration. In our solution, we employed qualification certification: registered doctors were from tertiary hospitals with years of experience in T1DM management. A test for doctors’ specialties may also be needed to ensure the qualifications of registered doctors.

### Comparison With Prior Work

The effects of current diabetes apps on T1DM are poor. No study has explored the reasons for this ineffectiveness from a user’s perspective, and very few diabetes apps have shared their methodology [23]; thus, app developers do not know how to choose a valid method. App development should be based on thorough knowledge of user needs [53]. Two studies designed diabetes apps by exploring users’ needs though in-depth interviews with young patients and their parents [54,55]. However, because the interviewees had never used diabetes apps, their understanding of diabetes apps was abstract, and they had difficulty describing their needs accurately. In addition, a purely qualitative study may not provide a comprehensive understanding of user needs. Castensoe-Seidenfaden et al first introduced a mixed-method study to design an app for improving self-management of young patients [56]. However, their quantitative and qualitative prestudies did not investigate patients’ and doctors’ perspectives of diabetes management apps. Our app design was led by diabetes experts. First, we conducted a quantitative survey to grasp the perspectives of patients and diabetes experts about diabetes apps from a macro level. Second, in-depth interviews with experienced patients supplemented and deepened the results of the questionnaire survey and gave us a better understanding of the problems of current apps and the need for a new diabetes app.

### Conclusions

Patient-doctor communication is the most important function of a diabetes app. A mobile app is the preferable method for patients to receive DSME compared with studying in a hospital, but apps should be integrated with HCPs rather than stand-alone. We advocate that doctors follow up with their patients using diabetes apps. Our mixed-method study combined qualitative and quantitative data to comprehensively and deeply explore why the effects of current diabetes apps in T1DM are poor and what T1DM patients need for a diabetes app from the user perspective, which provided meaningful guidance for our app design. This study has significance as a reference for the development of similar apps in the future.

## Appendix

Multimedia Appendix 1 Survey for diabetologists.

Multimedia Appendix 2 Survey for type 1 diabetes mellitus (T1DM) patients.

Multimedia Appendix 3 Interview guideline.

Multimedia Appendix 4 App functions and design.

## Abbreviations

DSME: diabetes self-management education
EMR: electronic medical record
HbA_1c_: average glycosylated hemoglobin
HCP: health care professional
IQR: interquartile range
RCT: randomized controlled trial
T1DM: type 1 diabetes mellitus
T2DM: type 2 diabetes mellitus
