# Supplementary material for: Exploration of Users’ Perspectives and Needs and Design of a Type 1 Diabetes Management Mobile App: Mixed-Methods Study
Source: JMIR Mhealth Uhealth. 2018 Sep 21;6(9):e11400. doi: 10.2196/11400 (PMC6231832; doi:10.2196/11400)
Supplement: Multimedia Appendix 1 [file mhealth_v6i9e11400_app1.pdf]

Perspectives of diabetologists concerning diabetes app

Name Gender Age

Tile：◎resident doctor ◎attending doctor ◎associate chief physician ◎chief physician

Hospital name：

Hospital degree：◎upper first-class hospital ◎middle first-class hospital ◎second-class hospital ◎second-class below

1.Have you ever down-loaded a diabetes app？

（1）No （2）Yes

2.Have you ever recommended diabetes apps to your patients？

（1）Yes （2）No

3. Which factors influence your recommendation of diabetes app to patients？（multi-choice）

（1）Not having information about diabetes apps

（2）The effectiveness of the apps was not evidence-based

（3）APPs are too many, I don’t know which is suitable

（4）Apps are useless

（5）Busyness （6）Others（please specify）________________________________

4.Have you ever used an app to manage patients with diabetes ？ **（if no, please jump to question 7）**

（1）Yes （2） No

5.Need patients pay for you when you manage their blood sugar ？（1）Yes （ 2 ）No

6. How many patients have you managed with diabetes app ？

(1) Less than 10 (2)11-50 (3) 51-100 (4) More than 100

7. Do you think app is useful for blood sugar control?

(1) Useless (2) A little useful (3)Useful (4) Very useful

8.Which do you think is the biggest obstacle for your use of apps to manage diabetes patients？（Single choice）

(1)How to charge (2) Limited time (3) Issue of legality (4)Patients’ distrust (5) Others （please specify）________________________________

9. Do you think busyness influenced you to manage patients with app？

(1) No (2) A little (3)Yes (4) Yes, very big

10. Do you think how to charge influenced you to manage patients with app？

(1) No (2) A little (3) Yes (4) Yes, very big

11.Do you think it is legal to use an app to guide patients’ medication？

（1）I don’t know （2） legal （3） illegal

12. Do you think patients’ distrust influenced you to manage patients with app？

(1) No (2) A little (3) Yes (4) Yes, very big

13.Which do you think are the reasons for invalidity of diabetes apps？（multi-choice）

（1）Patients can’t stick to using them

（2）Patients received little guidance from HCPs

（3）Diabetes education knowledge on apps were unsystematic

（4）App functions were incomplete

（5）Others（please specify）________________________________

14. Which do you think is the most important functions of app？（single choice）
（1）Diabetes diary（blood sugar, diet, exercise and medication records）
（2）Patient-doctor communication
（3）Diabetes education knowledge （4）Peer support
（5）Insulin dose calculator （6）Abnormal blood sugar reminder
15 Do you think feature of blood sugar record on app is important？
（1）No （2） A little important （3） Important （4）Very important
16. Do you think feature of diet record on app is important？？
（1）No （2） A little important （3） Important （4）Very important
17. Do you think calculation of calories and carbohydrate on current apps are accurate?
（1）Completely inaccurate （2）A little inaccurate (3) Accurate (4) Very accurate
18. Do you think it is important for doctors to participate in app？
（1）No （2） A little important （3） Important （4）Very important
19. Do you think it is important for diabetes nurse to participate in app？
（1）No （2） A little important （3） Important （4）Very important
20. Do you think it is important for dietitian to participate in app？
（1）No （2） A little important （3） Important （4）Very important
21. Do you think it is important for psychologist to participate in app？
（1）No （2） A little important （3） Important （4）Very important
22. Do you think diabetes education knowledge on app is important？
（1）No （2） A little important （3） Important （4）Very important
23. Do you think diabetes education knowledge on current diabetes apps are systematic？
(1) All are not systematic (2) Only a few are systematic (3) Most are systematic (4) All are systematic (5) I don’t know
24. Do you think peer support on app is important for patients？
（1）No （2） A little important （3） Important （4）Very important
25. Do you recommend an insulin calculator on app?
(1)I’m opposed to it (2) I don’t recommend it (3) I recommend it (4) I strongly recommend it
26. Do you think insulin calculators on apps are accurate?
(1) Completely inaccurate （2）A little inaccurate (3) accurate （4）very accurate （5）I don’t know
27. Do you think insulin calculators on apps are dangerous for patients？
（1） Not dangerous at all （2） A little dangerous （3） Dangerous （4） Very dangerous
28. Do you think abnormal blood sugar reminder on apps is important？
(1) No (2) A little important (3) Important (4) Very important
29. Do you think type 1 diabetes and type 2 diabetes need different apps？
(1) No (2) Yes
30. Which type of diabetes patients do you think need diabetes management app most?
（1）Type 1 diabetes（2）Type 2 diabetes（3）Gestational diabetes（4）Monogenic diabetic patients
31. Will you use an app to manage patients in the future？
(1)No （2）Maybe （3）Of course
32. Will you recommend diabetes app to your patients in the future？
(1)No （2）Maybe （3）Of course

33. How do you think of the prospects of the diabetes app？

（1）Bad （2）Just so-so （3）Good （4）Very good
