# Supplementary material for: Exploration of Users’ Perspectives and Needs and Design of a Type 1 Diabetes Management Mobile App: Mixed-Methods Study
Source: JMIR Mhealth Uhealth. 2018 Sep 21;6(9):e11400. doi: 10.2196/11400 (PMC6231832; doi:10.2196/11400)
Supplement: Multimedia Appendix 2 [file mhealth_v6i9e11400_app2.pdf]

Perspectives and needs of diabetes patients concerning diabetes app

Name： **gender：** □male □female **age：** _____years

Age of disease onset： _____years

Diabetes type： □type 1 diabetes □type 2 diabetes □gestational diabetes □I don't know

Treatment type： □ oral hypoglycemic agents □insulin injection □insulin pump □lifestyle intervention

Residence： ______province______city______county □ urban □ rural

Education： □ Primary school or below □ middle school □ high school □ university □ postgraduate

Occupation： □student□□ a civil servant or staff member □□ staff and workers of state owned enterprises □ employees of foreign enterprises / private / joint ventures □ private business owners or individual business households □retired □farmer □ unemployed □others（please specify）_________

Parents' education

Father□□primary school or below □□ middle school □□ high school □□ university □ □postgraduate

Mother □primary school or below □□ middle school □□ high school □□ university □ □postgraduate

Parents' occupation：

Father □ a civil servant or staff member □□ staff and workers of state owned enterprises □ □ employees of foreign enterprises / private / joint ventures □ private business owners or individual business households □retired □Farmer □ unemployed □others（specify）_________

Mother □ a civil servant or staff member □□ staff and workers of state owned enterprises □ □ employees of foreign enterprises / private / joint ventures □ private business owners or individual business households □retired □farmer □ unemployed □others（please specify）_________

Marriage： □unmarried □married □divorced

Annual income of family□□□less than 50000 yuan□□50001-100000yuan □100001-150000 yuan □□more than 150000 yuan

1.Do you know diabetes apps？**（if no, jump to question 25）**

（1）no （2）yes

2. Have you ever used a diabetes app?**（if no, jump to question 25）**

（1）no （2）yes

3.Who recommended your diabetes app？

（1）health care professionals （2）diabetes friend （3）media （4）I selected randomly because I did not know which one was best （5）others（please specify）_________________

4. How frequent do you use diabetes app?

（1）everyday （2）3-6 days a week（3）1-2 days a week （4）less than one time a week （5）less than one time a month（6）I hardly use it

5. Why you don't want to use your diabetes app？**（multi-choice）**

（1）complicated operations （2）useless （3）lack of time （4）cost （5）others（please specify）____

6. Do you think diabetes app is helpful for you?

（1）useless （2）a little helpful （3）helpful （4）very helpful

7.Does your app has a function of blood sugar record？**（if no, jump to question 10）**

（1）no （2）yes

8.Do you think manual input of blood sugar in your app is burdensome?？

（1）no （2）a little burdensome （3）burdensome （4）very burdensome

9. How frequent do you use diabetes app to record blood sugar?

（1）everyday （2）3-6 days a week （3）1-2 days a week （4）less than one time a week （5）less than one time a month（6）I hardly use it to record blood sugar

10. Does your app have a function of recording diet？**（if no, jump to question 12）**

（1）no （2）yes

11. How frequent do you use diabetes app to record diet?

（1）everyday （2）3-6 days a week（3）1-2 days a week （4）less than one time a week （5）less than one time a month（6）I hardly use it to record

12. Does your app have a function of recording exercise？**（if no, jump to question 14）**

（1）no （2）yes

13. How frequent do you use diabetes app to record exercise?

（1）everyday （2）3-6 days a week（3）1-2 days a week （4）less than one time a week （5）less than one time a month（6）I hardly use it to record

14. Does your app have a function of consulting health care professionals？**（if no, jump to question 19）**

（1）no （2）yes

15. Have you ever consulted health care professionals using diabetes app？**（if no, jump to question 19）**

（1）no （2）yes

16. Do you have to pay if you consult health care professionals using your app？

（1）no （2）yes

17. Do you think consulting health care professionals on app is helpful for you？

(1) Not helpful at all （2）a little helpful （3）helpful （4）very helpful

18. Why do you think consulting the health care professionals on app is helpless for you？

（1）Responses are not in time （2）They lack expertise （3）Communication time is too short （4）Others（please specify）______________________________________

19. Does your app have diabetes education knowledge？**（if no, jump to question 21）**

（1）no （2）yes

20. Do you think diabetes education knowledge on your app is helpful for you？

(1) not helpful at all （2）a little helpful （3）helpful （4）very helpful

21. Does your app have an insulin dose calculator？**（if no, jump to question 23）**

（1）no （2）yes

22. Do you think insulin dose calculator on your app is accurate？

（1）inaccurate （2）accurate （3）I don't know

23. Does your app have the function of abnormal blood sugar reminder？

（1）no （2）yes

24 Does your app have the function of blood sugar test reminder？

（1）no （2）yes

25. Which function do you think is most important for you if we design a diabetes app？**（Single choice）**

（1）diabetes diary（blood sugar, diet, exercise and medication record）

（2）consulting HCPs

（3）diabetes education knowledge

（4）peer support

（5）insulin dose calculator

（6）abnormal blood sugar reminder

（7）blood sugar test reminder

（8）others（please specify）____

26. Do you think diabetes diary（blood sugar, diet, exercise and medication records）on diabetes app is important for you？

(1) not important at all （2）a little important （3）important （4）very important

27. Do you think the function of consulting HCPs on diabetes app is important for you？

(1) not important at all （2）a little important （3）important （4）very important

28. Do you think diabetes education knowledge on diabetes app is important for you？

(1) not important at all （2）a little important （3）important （4）very important

29. Do you think the function of peer support on diabetes app is important for you？

(1) not important at all （2）a little important （3）important （4）very important

30. Do you think the function of insulin dose calculator on diabetes app is important for you？

(1) not important at all （2）a little important （3）important （4）very important

31. Do you think the function of blood sugar test reminder on diabetes app is important for you？

(1) not important at all （2）a little important （3）important （4）very important

32. Do you think the function of abnormal blood sugar reminder on diabetes app is important for you？

(1) not important at all （2）a little important （3）important （4）very important

33. Do you think you have to pay if you consult medical staffs using apps？

（1）no （2）yes

34. If you have to pay for your diabetes app, will you continue to use it？

（1）no （2）If effect is good, I will （3）yes, certainly

35. Do you want to have a good diabetes app to help you to manage your blood sugar？

（1）no （2）yes, I need （3）yes, I’m in great need of it
