# Supplementary material for: Exploration of Users’ Perspectives and Needs and Design of a Type 1 Diabetes Management Mobile App: Mixed-Methods Study
Source: JMIR Mhealth Uhealth. 2018 Sep 21;6(9):e11400. doi: 10.2196/11400 (PMC6231832; doi:10.2196/11400)
Supplement: Multimedia Appendix 3 [file mhealth_v6i9e11400_app3.pdf]

1 Have you ever received diabetes education program in hospital？why？

2 which do you prefer, studying on diabetes app or receiving diabetes education program in hospital? Why?

3 How do you control your blood sugar in your daily life? (including self-monitoring blood sugar, diet management, exercise management, insulin management ）

4 What functions does your APP have? What are the problems of these functions? Do you have any suggestions?

5 What suggestions and needs do you have if we design a diabetes app?
