# Supplementary material for: Exploration of Users’ Perspectives and Needs and Design of a Type 1 Diabetes Management Mobile App: Mixed-Methods Study
Source: JMIR Mhealth Uhealth. 2018 Sep 21;6(9):e11400. doi: 10.2196/11400 (PMC6231832; doi:10.2196/11400)
Supplement: Multimedia Appendix 4 [file mhealth_v6i9e11400_app4.pdf]

Multimedia Appendix 4: app functions and design

**App modules**	**Problems of current apps and patients' needs for a new app**	**app functions**	**suggestions**
patient-doctor communication	Distrust		Advocate doctors to follow up with their outpatients or inpatients
		Eligibility assessment: registered doctors are from tertiary hospitals with years of experience in T1DM management	
	Responses are not timely	Notifications are automatically sent to the patients or their doctors if there are new messages for them	
	inconvenient	Various types of communication modes: typing words, sending pictures, video chat and phone calls	
	Cost		Coverage of health insurance is needed
			Government subsidy policy is needed
Diabetes diary	Manual input of data is burdensome	Blood sugar results can automatically be transferred from glucose meter to our app by Bluetooth or GPRS	
		Patients can take photos of foods using the built-in camera if they have no time for detailed diet records	
		Daily step counts can be recorded automatically by obtaining step count data from step counter software in mobile phones	
	Food data-base is needed	Introduce calories and nutrients of foods	
		Calculates recommended intake of calories, carbohydrates, fat and protein automatically according	

		to patient's height, weight, and activity level	
	Lack of feed-back	Doctors view their patients' diabetes diaries and give tailored feedback	
		Set blood sugar targets. Off-target blood sugar alert.<br>If blood sugar is dangerous, a reminder message will be sent automatically to patient and his doctor's app.	
	Display is not as intuitive as paper diary	Blood sugar History graphs and statistics make blood sugars be clear at a glance	
		Diet, exercise, and medication can all be seen in the blood sugar display page, allowing a comprehensive analysis of the causes of blood sugar changes	
	Forget to test blood sugar	Blood sugar test reminder	
	Decline in disease alertness	Set a family member account to supervise the patients	
Diabetes education knowledge	Unsystematic; Unprofessional	Systematic T1DM education materials in our app are made by a multidisciplinary specialists panel	
	Avoid irrelevant knowledge interference	Education materials are specific for T1DM patients	
		Education materials are classified according to different categories, so that patients can select materials that they are interested in to learn.	
	Want to find interested materials easily	Search and navigation functions	
	Want Knowledge to be update in time	Renew latest progress in diabetes periodically	
		Set quizzes to test patients' diabetes knowledge level	

	Interaction is needed	Record the length of their study time about each diabetes education materials. Their doctors can see it and send tailored education materials to them	
	Tend to learn pop-up knowledge	New knowledge uses pop-up design	
	Different learning habits	Different modes of educational materials: videos, articles, PPT, short message and so on	
Peer-support	Exchange mode is inconvenient	Different modes of peer support: forums and group-chats	
		Add friends to chat privately	
		Notifications are automatically sent to patients if there are new messages for them	
	Avoid excessive information interference	Different categories of peer communities for patients to join in selectively	
	Peer leader is needed	Invite several experienced T1DM patients to each peer community as peer leaders	
	Privacy protection	Use nickname in the peer-support module	
Psychological module	Pay attention to patients' mental health	Scales such as self-evaluation of depression and anxiety are included in the app. If a score is abnormal, a message is sent automatically to the patient and his doctor to remind him to seek medical interference	Psychologists are needed to offer on-line psychological counseling
Electronic health records	Access to hospital EMR	Connect to hospital EMR to view their laboratory results, records of diagnosis and treatments	
